# A Rare Presentation of Zinner Syndrome

**DOI:** 10.7759/cureus.36220

**Published:** 2023-03-16

**Authors:** Shayan Mahapatra, Hovra Zahoor, Abraham M Quader, Jodi-Ann Chin

**Affiliations:** 1 Internal Medicine, Cleveland Clinic Florida, Weston, USA; 2 Internal Medicine, HCA Florida Orange Park Hospital, Orange Park, USA; 3 Internal Medicine, Edward Via College of Osteopathic Medicine, Spartanburg, USA

**Keywords:** developmental anomaly, renal agenesis, seminal vesical cyst, ejaculatory disorders, zinner’s syndrome

## Abstract

Zinner syndrome is a rare developmental anomaly of the distal Wolffian duct. It is characterized by a triad of unilateral renal agenesis, cysts in the ipsilateral seminal vesicle, and ipsilateral obstruction of the ejaculatory duct. While some patients are asymptomatic and diagnosed incidentally, other patients may present with symptoms related to obstructed ejaculatory ducts and seminal vesicle cysts. We report a unique case of a 32-year-old male who presented with pelvic pain for three days.

## Introduction

Zinner syndrome is a rare congenital urogenital developmental anomaly. The embryologic abnormality develops before the 7th gestational week in the distal portion of the mesonephric or Wolffian duct. This congenital maldevelopment is characterized by a triad of renal agenesis (unilateral), ipsilateral obstruction of the ejaculatory duct, and cysts in the ipsilateral seminal vesicle. Patients may present with dysuria, increased urinary frequency, painful ejaculation, and epididymitis (symptoms related to seminal vesicle cyst and ejaculatory duct obstruction). However, some patients are asymptomatic and diagnosed incidentally [1]. Here, we present a unique case of a 32-year-old male who presented with the rare clinical feature of acute onset pelvic pain and was eventually diagnosed with Zinner syndrome.

## Case presentation

A 32-year-old male with no significant past medical history presented to the emergency department with complaints of pelvic pain for the last three days. He reported associated symptoms of dysuria and difficulty urinating but denied any urgency, frequency, painful ejaculation, or hematospermia. He reported being sexually active but had no diagnosed history of infertility. A physical exam revealed mild suprapubic tenderness.

**Figure 1:**
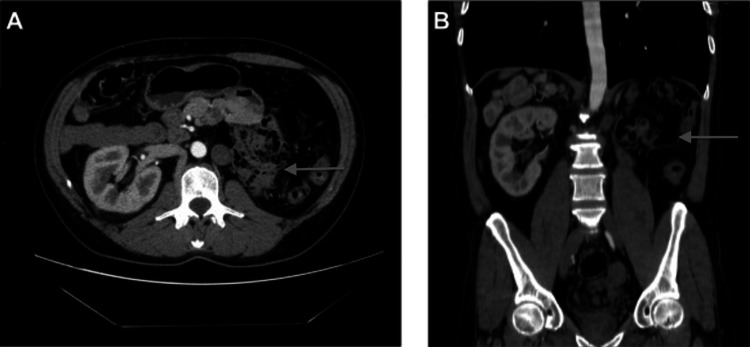
(A) Axial CT imaging with left renal agenesis. (B) Coronal CT imaging with left renal agenesis.

On work-up, complete blood count and comprehensive metabolic panel were within normal limits. Urinalysis was unremarkable. Ultrasound was negative for testicular torsion. Computed tomography (CT) abdomen/pelvis revealed left renal agenesis (Figures 1A, 1B).

**Figure 2:**
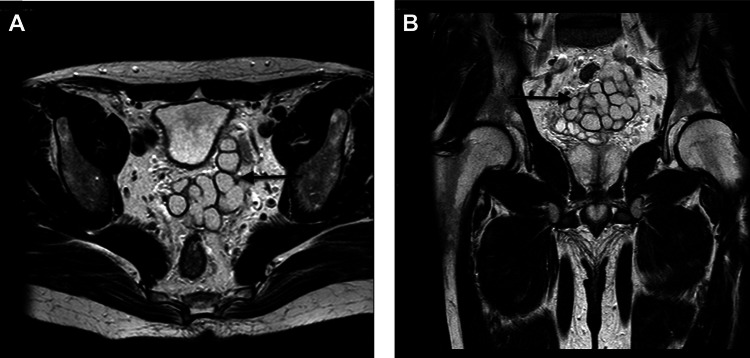
(A, B) MRI abdomen/pelvis revealed multiple cystic hyperintense structures in the region of the right seminal vesicle near the bladder base.

Magnetic resonance imaging (MRI) of the pelvis showed the right kidney to be normal with a normal collecting system and no calculus. There was left renal agenesis noted however with a 7.6 cm left seminal vesicle cyst communicating with a left retroperitoneal structure suggesting ectopic ureteral insertion (Figures 2A, 2B).

**Figure 3:**
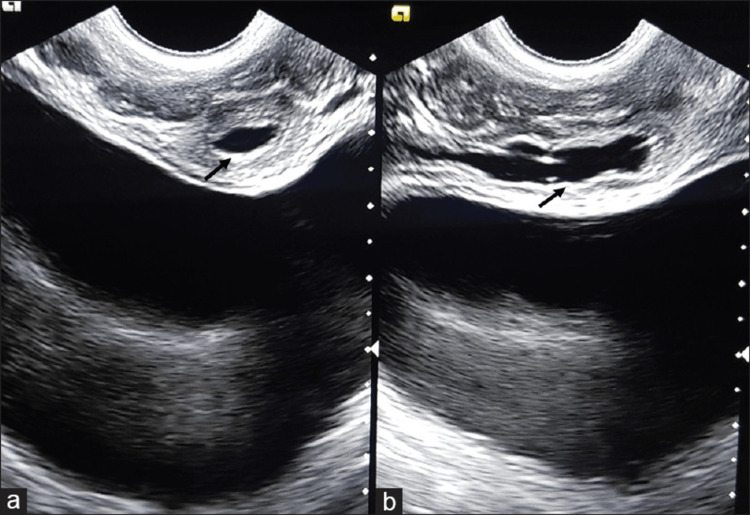
(A, B) Trans-rectal ultrasound image showing cystic structure in the seminal vesicle region.

There was non-visualization of the left ductus deferens. Cystoscopy and retrograde pyelogram were performed which showed a cystic mass, trabeculations and cellules on the bladder indicating bladder outlet obstruction. Transrectal ultrasound revealed similar findings (Figures 3A, 3B).

The patient was treated with robotic left seminal vesicle excision with excision of the left ureter and atrophic left kidney. Intraoperative findings confirmed the diagnosis of Zinner syndrome.

## Discussion

Zinner syndrome was first described in 1914 [2]. Since then, there have been few reports of the condition. The literature review revealed an incredibly low incidence. The most widely acknowledged study came from Taipei in 1990, where the incidence of seminal vesicle cysts with ipsilateral renal dysplasia in children was found to be 0.0046% [3]. The syndrome is distinguished by the triad of unilateral renal agenesis, seminal vesicle cysts, and ipsilateral ejaculatory duct obstruction. These characteristics are explained by the close embryological relationship between the ureteric bud and the male reproductive system that takes place in early development. Between the sixth-eighth gestational week, the distal mesonephric duct and the ureteric bud separate, and the ureteric orifice then migrates to the metanephric blastema. At the same time, testosterone and anti-Mullerian hormone influence the distal part of the mesonephric duct to form a structure called the hemitrigone. The hemitrigone goes on to form the urethra up to the external sphincter, bladder neck, seminal vesicle, vas deferens, ejaculatory ducts, epididymis, paradidymis, and the appendix of the epididymis [4]. Sequentially, the ureteric bud is formed by the metanephric blastema as it secretes growth factors during the fourth-sixth gestation period. The ureteric bud proliferates and fuses with the metanephric blastema to form the primitive kidney before undergoing epithelial transition [5]. Mutations of the metanephric blastema, disruption of retinoic acid signaling, or any other undescribed disturbances can cause renal agenesis or hypoplasia. Equally, disruption of the process of separation of the ureteric bud from the distal mesonephric duct can cause ejaculatory duct atresia and seminal vesicle obstruction resulting in the accumulation of secretions. This can result in cystic dilation and manifests as the seminal vesicle cysts that characterize Zinner syndrome. The latter attributes to azoospermia and oligozoospermia manifests as infertility in the third or fourth decade of life in affected males [6]. Clinical manifestations may vary and are often associated with the time of insult during embryogenesis. Errors occurring before the seventh gestational week have a higher association with renal agenesis.

A pooled analysis published in 1998 by Van den Ouden sought to provide a review of common symptoms for clinical reference. This study found the most common presentation to be dysuria (37%), followed by increased frequency (33%). Perineal pain was found in 29% of patients, epididymitis in 27%, pain after ejaculation in 21%, and scrotal pain in 13%. 17% of patients suffer from infertility [1]. An updated pooled analysis published in 2021 found the most common symptoms to be urinary frequency, urinary urgency, dysuria, and perineal pain. This study found an association between symptoms and cyst size [3]. Increased cyst size is associated with pelvic and perineal pain as pressure effects can affect surrounding structures, although this clinical feature is rare. Asymptomatic patients with seminal vesicle cysts were found to have cysts measuring less than 5 cm. These cases were reported to be discovered incidentally during a routine digital rectal examination or unrelated imaging. Bladder and colonic obstruction were seen in cysts larger than 12 mm [6]. The patient illustrated in this article had a cyst measuring 7.6 cm which likely was the cause of his new onset of pelvic pain.

Appropriate imaging modalities are essential in the evaluation of complex development anomalies of the urogenital tract. Differential diagnoses, such as ejaculatory duct cysts, ureterocele, acquired seminal vesical cysts, or prostatic utricle cysts, could be ruled out based on imaging findings and patient history. The most frequently used imaging modalities for diagnosis were magnetic resonance imaging (MRI) (67.8%) and ultrasonography (65.0%) [3]. On ultrasound, the ipsilateral kidney will be absent, and the seminal vesicle cyst is usually seen as a pelvic mass that is anechoic and with an irregular, thick wall [7]. Obstructed ejaculatory ducts can also be detected on Sonography. Further and more detailed evaluation can be done with transrectal ultrasound (Figure 3). However, only MRI or CT can outline the anatomy accurately thereby helping identify Zinner syndrome and also differentiate it from other malformations. CT findings are of a retro vesicular cystic mass superior to the prostate gland and associated ipsilateral renal agenesis. MRI is the modality of choice to evaluate anomalies of the mesonephric duct and to provide accurate identification and location of the cyst. Like other cystic masses in the body, they appear hypointense on T1W depending on protein content and hyperintense on T2W images. MRI is the only currently available modality that can differentiate seminal vesicle cysts from other cystic masses in the pelvis by the presence of a convoluted tail connecting the cystic anomality to the seminal vesicle [7]. MRI can also identify ectopic ureteric orifices, unlike other modalities. Most patients are asymptomatic and do not require intervention. For the symptomatic cases, only surgical resection of the cyst and the involved seminal vesicle is known to be 100% effective. Conservative transrectal aspiration is often tried by practitioners but carries a high risk of recurrence (47.4%) and infection with a success rate of 30%. Additionally, studies have shown multiple complications with repeat attempts after initial failure [1]. Until recently, only open transabdominal or trans perineal vesiculectomy was performed. Robotic-assisted approaches have recently been developed that offer a minimally invasive approach with better visibility, shorter hospital stays, fewer pre/post-operative complications, and faster recovery. To our knowledge, only six cases of Zinner syndrome have been described in the literature as treated by robotic surgical resection [8]. The patient in this article was also treated with robotic left seminal vesicle excision with excision of the left ureter and atrophic left kidney. Surgical removal of the anomalies is described to be successful in most cases. The comorbidities and complications experienced by patients with Zinner syndrome have not been explored extensively in the past. In one review, tumors arising from the urogenital system occurred in 4.4% of patients studied [3].

## Conclusions

Zinner syndrome is a rare congenital urogenital developmental abnormality. The incidence is low and clinical presentation commonly includes urinary symptoms, epididymitis, and painful ejaculation. Our case illustrates an atypical presentation of Zinner syndrome and highlights the importance of holding a high index of suspicion in appropriate clinical settings. A careful review of imaging is crucial as it is the primary modality of diagnosis and misdiagnosis can lead to adverse outcomes like infertility at a young age.
